# Barnes’ Obstetric Operations

**Published:** 1870-07

**Authors:** Volta Reeke


### Review.

*Obstetric Operations, including the Treatment of Hæmorrhage.* By Robert Barnes, M. D., Lond., F. R. C. P.; *with Additions*, by Benjamin F. Dawson, New York City. *First American Edition.*

This book was handed us a few days since for perusal, but our time has been so limited, that we have been forced to run through it in a very superficial, and therefore unsatisfactory, manner; yet it is one of such transcendent merit, we could not refrain from devoting a few moments to speak of its many excellencies, and to give our just mead of praise in favor of its distinguished author.

There exists certainly no medical work in the English language, treating of the various and complicated operations of midwifery; defining so clearly and truthfully the conditions calling for interference; and explaining in such practical terms the mechanism of abnormal labors,—to compare with this; and we can safely affirm that there can be found no surer friend, nor safer guide to the obstetrical physician, and especially to the younger and less experienced members of our profession, who may wish to make this branch an especial study.

The first four or five chapters give us a selection and description of the instruments needed to make up the bag of the accoucheur; their modes of application, and the unnatural states demanding their uses—the lever as a valuable aid in facilitating labors with head presentations; the forceps, not only as an instrument of extraction and compression, but possessing immense powers of leverage, etc. And here he interposes his objections to the short single-curved forceps, as being difficult of introduction, being obliged to seize the head in its transverse diameter, regardless of position or the curves of the pelvis; as tending to lacerate the perinæum even in the hands of the most skillful; almost certain to bruise or tear the edge of the sciatic nerve by the posterior or sacral blade, thus giving rise to more or less protracted paralysis of the leg; and lastly, by pressing on the portio dura as it emerges from the temporal bone, very often paralyzing the side of the face, leaving a staring eye, and inability to suck. His objections are well taken, and his arguments in favor of the double-curved long forceps, sustained by a large and ripe experience, will carry conviction to every mind. This latter instrument is applied on an entirely different principle; adapting itself to the curves of the pelvis, one blade in each ilium, it grasps the head in any position, and by assisting the action of the uterus, *not supplanting it*, gradually extracts from brim to outlet. A description is given of its application to first-coming and after-coming heads, every step of the operation of extraction fully explained, and he winds up by saying that the mortality from its use is *nil*, death occurring only when it is applied too late, and that, therefore, the argument of *post hoc, ergo propter hoc* cannot avail here. Next in order, we arrive at face presentations, one of the best chapters in the book, where the subject is thoroughly discussed in all its bearings, and its importance will be fully recognized, when we state, in conjunction with all physicians of large practice, that there can occur few complications, engendering such prolonged labors, so painful, and often dangerous to the mother, and so wearisome and harassing to the obstetrist. Chapters VIII and IX are devoted to the multiple agencies producing dystocia, from the various deformities of the pelvic bones, to every faulty condition of the soft parts, and then we come to version, with a highly interesting philosophical explanation of abnormal presentations requiring it, and the bi-polar method of assistance, its great utility in rectifying mal-positions, cephalic and pedalic, and the great range of its application. The bi-polar version was first practiced by Wigand, d'Outrepont and others, with both hands externally applied, whereas the present method, one hand internal and one external on the uterine tumor, was fully elaborated and introduced by Dr. Braxton Hicks, and is now considered of such value, that many cases before impossible to version, can be readily managed by its aid. We here call to mind, witnessing many years ago, in cases of protracted labor, the "Old Grannies" of the plantations, often hop astride their lying-in women, and by a combined sliding, jogging and pressing motion, like a boy on his hobby-horse, endeavor to hasten the expulsion of the fœtus, while the recipients of such gratuitous favors would yell and howl in their agony, alternating with expressions of religious fervor and prayer, not unlike, however, their white congeners; for, in times of pain and peril do we not all become so very good? as explained so racily in that old well-known couplet:

> "When the devil was sick, the devil a monk would be,
> When the devil was well, the devil a monk was he."

He places the limit of turning at 3.25″ to 3.75″ conjugate diameter, when at the latter point it comes in competition with the forceps, and below the former, begins to encroach upon the domain of craniotomy, etc. In performing version, he recommends taking the knee to the foot, and always the *farther*, or *opposite* one, as traction upon it rotates the fœtus upon its long axis, and it comes down more easily.

Craniotomy and cephalotripsy are taught *in extensô*, presenting, however, no new features, until we reach the author's method of embryotomy, performed by his "wire écraseur," manufactured by

Weiss, and endowed with an archimedean screw and windlass. The application of this instrument is easy, the whole force of the operation is expended upon the child, thus avoiding the contusions and lacerations of the mother, and the head can be so reduced—by removing an occipital segment, and also a facial—as to pass an antero-posterior diameter of 1.00″; then the remainder, by lopping off the arms, and eviscerating the chest and belly. He is thus enabled to spare the mother in many instances the dangers of the cæsarean section. In regard to this last, he views it both in its moral and material aspects, leaning ever to the side of humanity, handling the mother with all the tenderness of a noble and generous nature, and by his sound logic sweeping away many of the old guides and landmarks of sacrificial midwifery. He takes the limit at 1.75″, and generally in those cases of osteomalacia, where the bones will not open up under pressure, and where the transverse and diagonal diameters offer insufficient accommodation for a narrow conjugate. He believes it is rarely called for in rickety pelvis; occasionally when craniotomy may have failed; in some cases of rupture of uterus; malignant disease; death of mother, etc. The induction of premature labor is presented to us as a very important subject, and necessitated, of course, by those conditions where a fully developed child could not be brought into the world without a sacrifice of life. In cases of contracted pelvis, the conjugate diameter 2.50″, the development should not exceed 250 days, and we should rather run the risk of a non-viable child; and with still smaller diameter, the operation becomes more imperative, though the birth may require extraneous interference. That a child may be viable, and stand some chance of surviving, he reckons the pregnancy from the day after the last menstrual epoch, counts 230 days, and adds 20 more as a margin of safety, thus leaving one full calendar month for the complete growth of fœtus. Among the means of inducing labor prematurely, he describes the many modes of cervical dilatation, from the vaginal douche of Kiwish; the injections *in uterô* of fluids by Lazarawitch; of gases and of air; tents; irritation by various "diastaltic persuaders;" and finally, best of all, the immediate method by his "hydrostatic dilators." In cases of great emergency, dilatation and delivery can be secured anywhere from one to six hours, the caoutchouc dilators acting upon the principle of the "bag of waters," exerting a steady equable pressure, elastic and sure to relax the most rigid os. The author's method of procedure challenges our attention. He divides it into two stages; the first, the *provocative;* the second, *accelerative;* and when the operation is determined upon, he introduces at night an elastic bougie seven or eight inches into the womb, and tucks away the outside portion in the vaginæ. Next day, finding the cervical canal somewhat open, and evidences of commencing uterine action, he proceeds to the second or *accelerative* stage, punctures the membranes, and leaves the rest to Nature; but in those cases where complications arise demanding immediate action, dilates with his caoutchouc bags, and, if necessary, turns so as to complete the delivery soon as practicable.

Placenta prævia comes next into notice, and forms the most important chapter of the book. In this he explains his physiological doctrines of development and implantation; the philosophy of hæmorrhage; and controverts the old fashioned ideas of treatment, showing their weakness and fallacy; for instance, the method prescribed of passing the hand and detaching the placenta *in totô*, he demonstrates its utter impracticability, since the placenta in the greater number of cases extends higher than the meridian of the uterus, very often reaching as high as the fundus. the fingers not being long enough to extend half way to its farther margin, and the cervix being generally too rigid and undilatable to permit the passage of hand in cavity of womb.

*A priori*, it is assumed that total detachment stops bleeding; this is partly true, and partly fallacious; for if the placenta is thrown off *spontaneously* hæmorrhage ceases, but it does not justify the belief of arrestation upon *artificial* separation. There exists a fundamental physiological distinction between the two cases. In the first instance *spontaneous* detachment is due to the contractile power of the uterus, the hæmorrhage being arrested by the same agency; whereas, if pulled off *artificially*, there being defective uterine contraction, bleeding continues. The chief points in placental attachment are illustrated by a diagram, dividing the internal uterine surface into three zones, fundal, meridional, and cervical; the first, the seat of *safe* attachment; the second, *comparatively safe*, but may lead to obliquity of womb, oblique position of the child, lingering labor, and thus dispose to retention of placenta, and *post partum* hæmorrhage; the cervical, the place of *dangerous* attachment, and all implantations here, whether it be the encroachment of a small portion, or whether the entire mass, is liable to previous dislodgment. This lower polar circle is therefore the physiological line of division between prævial and lateral placentæ, *below* which you have spontaneous detachment and hæmorrhage, *above* which, neither. Having entered these points, the treatment is evident; puncture the membranes to allow the uterus to exert a proper pressure; effect dilatation with the "hydrostatic bags," if it becomes necessary; and should hæmorrhage still continue, then introduce one or two fingers and detach that portion of placenta within the orificial zone; still further hasten by the oxytocics, or resort to version. The hæmorrhage resulting from placenta prævia is called by Rigby the "unavoidable," to distinguish it from his "accidental," occasioned by detached placenta during gestation. The differentiation of the accidental is not always an easy matter, being determined by subjective symptoms generally; but, if occurring, whatever the cause, it calls for immediate delivery, by the methods above enumerated, at the same time keeping up your patient by warm cordials, food, etc., etc.

As to the *post partum* hæmorrhages, whether from retained placenta due to unequal uterine contraction, or to adhesion from diseased conditions, or to accumulation of clots, etc., etc., we have little to say, except to notice one simple and invariable rule; that is, to control them, the *uterus must be fully emptied of its contents*, not only by every means possible to induce contraction, but by the introduction of the hand, and even by the use of the wire ecraseur should retained portions, or cotyledons, be the causation, and if then bleeding should persist, the injection, repeated if necessary, of the per-chloride of iron—1 to 3 of water. Such complications as fibrous tumors, polypi, inversion, etc., are to be managed upon general principles, though in regard to the reduction of inversions by pressure and manipulation, he says, in passing back the womb, remember the pelvic axes, and when you reach the sacral promontory, pass the organ to *one side*. The operation of Dr. T. G. Thoms is here introduced by the American editor, as being novel, and the only means we may possess, when all others have failed. *Prima facie* it seems a formidable one, yet, if the patient is prepared by the exhibition of opiates several days before, as recommended by the celebrated Dr. Todd, in all operations involving the peritoneum, there exist very strong reasons to predict a safe recovery. He cuts a few inches along the medion line above the symphisis pubis, into abdominal cavity, finds the inverted neck, introduces a steel instrument like a glove stretcher, dilates, and by the taxis returns the womb to its normal position.

Transfusion is spoken of very cleverly, and he looks upon it, *in futurô*, as a powerful agency in the hands of professional men to restore life, where excessive bleedings have taken place, etc. Without commenting upon the various methods and fluids to be employed, we may state that this operation will prove more uniformly successful where defibrination is practiced, and this process has been very ably demonstrated by Prof. Freer, of this city, not many months back.

The appendix treats of the difficulties of twin labors—head-locking and hæmorrhage—dorsal displacement of arm (as a rule due to the *nimia diligentia* in midwifery—an ugly complication, and, if not reduced, requiring operative interference); and the birth of monsters. There are no points to be particularly dwelt upon in this chapter, but what may be gleaned from any of the standard works, and in closing this analysis, we feel justified in giving this excellent treatise our most positive endorsement. A book that should be in the hands of every practitioner; clear and succinct in its statements; logical in its deductions; of pure diction, and thoroughly intelligible in its descriptions; filled with valuable facts coming from a man highly cultivated, of remarkable diagnostic powers; excellent judgment; and so humane and conservative that he should merit the thanks and gratitude of every member of our profession.

The American editor, with a modesty characteristic of true worth, has kept himself in the background, making few additions, and those from the best sources, and always *à propos* of the subject, and hence sparing us—for which he cannot be too highly commended—the insertion of prosy and lengthened extracts, generally so tedious and worthless, and only serving to interrupt the harmony of the original text.

"VOLTA REEKE."

